# Study Protocol: insulin and its role in cancer

**DOI:** 10.1186/1472-6823-7-10

**Published:** 2007-10-22

**Authors:** K Harish, M Dharmalingam, M Himanshu

**Affiliations:** 1 Department of Surgical Oncology, M. S. Ramaiah Medical College & Hospital, Bangalore 560054, India; 2 Department of Endocrinology, M. S. Ramaiah Medical College & Hospital, Bangalore 560054, India

## Abstract

**Background:**

Studies have shown that metabolic syndrome and its consequent biochemical derangements in the various phases of diabetes may contribute to carcinogenesis. A part of this carcinogenic effect could be attributed to hyperinsulinism. High levels of insulin decrease the production of IGF-1 binding proteins and hence increase levels of free IGF-1. It is well established that bioactivity of free insulin growth factor 1 (IGF-1) increases tumor turnover rate. The objective is to investigate the role of insulin resistance/sensitivity in carcinogenesis by studying the relation between insulin resistance/sensitivity and IGF-1 levels in cancer patients. We postulate that hyperinsulinaemia which prevails during initial phases of insulin resistance (condition prior to overt diabetes) increases bioactivity of free IGF-1, which may contribute to process of carcinogenesis.

**Methods/Design:**

Based on our pilot study results and power analysis of the same, we have designed a two group case-control study. 800 proven untreated cancer patients (solid epithelial cell tumors) under age of 50 shall be recruited with 200 healthy subjects serving as controls. Insulin resistance/sensitivity and free IGF-1 levels shall be determined in all subjects. Association between the two parameters shall be tested using suitable statistical methods.

**Discussion:**

Well controlled studies in humans are essential to study the link between insulin resistance, hyperinsulinaemia, IGF-1 and carcinogenesis. This study could provide insights to the role of insulin, insulin resistance, IGF-1 in carcinogenesis although a precise role and the extent of influence cannot be determined. In future, cancer prevention and treatment strategies could revolve around insulin and insulin resistance.

## Background

The association of diabetes mellitus and cancer has been reported more than 100 years ago [1]. Population studies have shown increased evidence of this association. Diabetes has been recognized as a risk factor for development of breast, endometrial, colorectal and pancreatic carcinomas [2]. Breast, endometrial, colorectal and pancreatic carcinomas are best studied with regard to their association with diabetes/insulin resistance. Population studies have shown that the effects of diabetes mellitus on colorectal cancer may be mediated through mechanisms ranging from increased colonic transit time to hyperinsulinaemia. In relation to the latter, at least in the early phase of development, type 2 diabetes mellitus is associated with increased circulating insulin concentrations [3,4]. A large cohort study concluded that diabetes is associated with a modestly increased risk for endometrial cancer among women [5]. Future research, particularly prospective studies with biological samples, could be very helpful in answering questions aimed at clarifying these mechanisms [5]. Data suggest that type 2 diabetes might be associated with up to 10–20% excess risk for breast cancer and that it could also have detrimental effects on the natural history, diagnosis, and treatment of breast cancer [1,6].

In the past few years both laboratory investigations and population studies have provided some circumstantial evidence that insulin growth factor (IGF) physiology influences cancer risk [7]. Insulin resistance is the underlying pathology of type 2 diabetes mellitus and is a component of metabolic syndrome [8,9]. Over the years, prospective studies have shown that metabolic syndrome and its consequent biochemical derangements may contribute to carcinogenesis. Several studies implicate hyperinsulinism, a condition that prevails prior to the onset of diabetes (part of metabolic syndrome) as candidate mediator in carcinogenesis [10]. Studies have revealed that high levels of circulating insulin decreases levels of insulin like growth factor binding protein 1 and 3 (IGFBP 1,3). Thus free IGF-1 levels increase in circulation [11,12]. Another aspect of IGF physiology is the IGF signaling. In this signaling process, IGF-1 receptor is a predominant factor and is crucial for tumor transformation and survival of malignant cell. It has comparatively less role in normal cell growth [13]. Thus role of IGF-1 in promoting carcinogenesis and its prognosis is well established. Over the recent years, IGF-1 physiology has been widely studied. The IGF system, comprises of insulin-like IGF-I, IGF-II, and IGFBPs. IGF-1 as a growth factor plays a dominant role over IGF-2 and hence is widely studied. Until recently, growth hormone was the only known stimulant of IGF-1 production. Different lines of evidence suggest that the IGF/IGFBP system may be regulated by factors other than growth hormones. States of nutritional deprivation, such as starvation and protein caloric under-nutrition and type 1 diabetes mellitus, in animal models have long been known to influence the production of IGFs [14-16]. Various studies have shown that resistance to insulin action, as found in diabetic patients, has been associated with an increase IGFBP-3 protease activity, there by reducing IGFBP-3 levels [17]. Also, Insulin increases IGF-I bioavailability through IGFBP-1 suppression [14,18,19]. Thus there is ample evidence to suggest that insulin resistant states increase free IGF-1 levels.

Medical literature suggests that well controlled biochemical and genetic studies are required to establish the link between insulin, IGF-1, diabetes and cancer. We propose to investigate the role of hyperinsulinemia in carcinogenesis.

The primary objective of the study is to investigate the role of insulin resistance or sensitivity in carcinogenesis. This is done by studying the relation between insulin resistance/sensitivity and IGF-1 levels in cancer patients. The secondary objective is to study the above mentioned association with organ specific cancers if possible.

Carcinogenesis is multi-factorial. The metabolic and genetic derangements that take place during carcinogenesis may be induced by carcinogens and inherited genetic factors. The role of either could be variable in a given case. We hypothesize that people with insulin resistance are at risk of developing cancer due to high levels of circulating IGF-1. Such a risk would increase if other such factors are prevalent/acquired. A person with high levels of IGF-1 may be predisposed to cancer and his/her risk of developing cancer would increase with the presence of other such factors. Cancer being a non-communicable disease, with multiple risk factors, modifiable risk factors are very few. Controlling hyperinsulinaemia would modify one major risk factor. IGF-1 levels in body change with age. In addition, risk factors for cancer and carcinogen exposure increase with age [20]. Thus age and known carcinogens could be major confounders.

In a pilot study conducted at our centre (unpublished data) to test the above hypothesis we found a strong trend towards significance in patients under the age of 50. Genetic factors play a major role in carcinogenesis in young patients (below 50 yrs). Hence IGF-1 and insulin may play a larger role in patients below 50 years. Thus we limit our study to cancer patients below the age of 50.

IGF-1 has high bioactivity on epithelial cells. Thus their role in carcinomas is of significance rather than other types of malignancies. Hence we limit our study to carcinomas of breast, GIT, liver, prostate, uterus, cervix and ovaries.

## Methods/Design

### Structure of study

This is a two-group, prospective, case-control study. First group shall comprise of newly diagnosed cancer patients, for whom treatment has not been initiated. The second group shall consist of subjects from normal population. Fasting blood sugar (FBS) shall be estimated for all subjects in both the groups. This is to rule out undiagnosed diabetes mellitus. A FBS level above 126 mg% is diagnostic of diabetes [21]. Fasting insulin level and IGF-1 level will be assayed in the same blood sample for all subjects.

### Sample size

Based on an unpublished pilot study results (conducted on 63 patients) 761 patients and 191 controls are required to expect a study power of 80 %. We plan to recruit 800 patients and 200 controls. Controls would be age and sex matched to that of the patients.

### Data and Sample collection

All subjects will be recruited from out patient division of M.S. Ramaiah Memorial Hospital, Bangalore, India and Cancer Institute, Chennai, India. Patients who are below age of 50, with carcinomas of breast, GIT, liver, prostate, uterus, ovaries and cervix shall be recruited. The exclusion criteria are; patients who have undergone treatment for cancer. Therapies for cancer including surgery and chemotherapy alter IGF-1 levels, growth factors and immune mediators. Patients on palliative therapy due to very advanced cancer and malnourished patients will also be excluded as these patients will have low IGF-1 levels due to cachexia [20]. Malnourishment will be assessed by Subjective Global Assessment (SGA) method [22]. Patients with endocrine related diseases, multiple endocrine neoplasia syndrome will also be excluded as IGF-1 is a secondary hormone and can be influenced by major hormone imbalances. Patients with diabetes mellitus (Type 1 and 2) will be excluded as such patients can have low IGF-1 levels due to altered nourishment. Patients with chronic liver diseases other than cancer will also be excluded as liver is a major producer of IGF-1. All patients with proven tobacco related cancers will be excluded.

Group 2 consists of subjects from normal population. Subjects without any chronic or acute diseases will be considered. Subjects under treatment for any medical diseases, subjects who are malnourished or subjects who have family history of cancer or subjects who have body mass index greater than 25 will be excluded. All the above factors affect IFG-1 levels.

After obtaining an informed consent from subjects of both groups, a detailed, relevant clinical history will be documented. A 5 ml fasting venous blood sample (by standard venous puncture) from all the recruited patients will be drawn. Free IGF-1, fasting blood sugar and fasting Insulin assays will be estimated on all samples.

### Laboratory Assays

8 hour fasting plasma blood sugar will be done by glucose oxidase – peroxidase (GOD-POD) method. Fasting insulin will be assayed by (Radioimmunoassay) RIA method using commercially available insulin RIA kit supplied by Diagnostic Systems Laboratories Inc., Texas. IGF-1 will be assayed by Immunoradiometric assay (IRMA) method using a commercially available IRMA kit supplied by the same company.

### Analysis

Homeostasis model assessment-insulin resistance (HOMA-IR) method will be used to calculate insulin resistance/sensitivity [23]. The same will be associated with IGF-1 levels using various statistical tools as mentioned below.

### Data Management

All data elements recorded during the study period will be entered and validated in Microsoft Excel. All variables and changes will be transferred into the Satastical Package for Social Sciences (SPSS), SYSTAT™ statistical software, summarized (Counts, Minimum, Maximum, Mean, Median, Standard Deviation) within each treatment group, transferred to the statistical report and then reformatted. Suitable graphs will be generated to depict the changes in key efficacy parameters and then transferred to the statistical report.

### Statistics

#### Descriptive Statistics

Descriptive statistics for each numerical variable will be summarized as the frequency, percentage, mean, median and standard deviations for all the subjects at each time interval in each study group.

#### Statistical methods

Chi-square test and Fisher exact tests will be used to analyze the frequency data, Student't' test (two-tailed) will be used to test the significance of study parameters between case and controls. Analysis of variance/Kruskal Wallis test will be used for within group analysis. Any other suitable statistical techniques will be used to find the effect of study parameters.

#### Control of Type -1 Error

All statistical tests will be conducted at the 0.05 alpha levels, meaning that P ≤ 0.05 will be considered "nominally significant". The Adjustment for multiple tests will be done by applying Bonferroni correction to the p-values. The p-value for the comparison of the primary efficacy endpoints between Cases and Control group will be considered to be conclusive.

#### Software

The Statistical software namely SPSS 11.0 and Systat 8.0 will be used for the analysis of the data and Microsoft word and Excel shall be used to generate graphs and tables.

#### Sub-Group Analysis

A similar analysis will be carried out on organ-specific cancers like breast, colon and rectum, depending on number of patients recruited.

### Ethical Issues

The study has been approved by Ethical Review Boards of all centers involved in the study. (Approval Ref – MSRMC/ERB/2007/06-01 dated 12^th ^June 2007).

### Informed Consent Process

The investigators shall provide to the subjects, all the study related information in a language desired by the subject and at a level of complexity that is understandable to the subjects and the guardians. Prior to the subjects' participation in the study, the written informed consent form shall be signed and dated personally by the subject, the guardian, the investigator and impartial witness.

### Interim Analysis

Two interim analyses shall be carried out. First analysis will be done once 30 % of patients are recruited. The second analysis will be done once 60 % of them are recruited. Recruitment will stop if the study attains positive significance at end of interim analysis.

## Discussion

Since carcinogenesis is multifactorial, various external carcinogens, genetic factors and metabolic derangements contribute to carcinogenesis. Diabetes mellitus type 2 and its associated metabolic derangements have been studied for their role in development of cancer. Hyperinsulinaemia ensues in early phase of insulin resistance which is responsible for variety of metabolic derangements. Hyperinsulinaemia has also been implicated as a candidate mediator in carcinogenesis [10].

High levels of insulin decrease binding proteins of IGF-1 [14,18,19]. IGF-1 signaling has been highly implicated in development of cancers, especially epithelial cell cancers (carcinomas). The IGF-1 signaling system plays a crucial role in tumor transformation, malignant cell turn over and survival of cancer cells. Such roles in normal cell growth are comparatively less [13,24,25]. The insulin IGF-1 system relationship and role of IGF-1 in carcinogenesis have been independent lines of investigations. However, well controlled studies in humans are essential to study the link between insulin resistance, hyperinsulinaemia, IGF-1 and carcinogenesis.

We hypothesize that hyperinsulinaemia which marks the early phase of insulin resistance could decrease IGFBP production. This results in increase in free IGF-1 in blood. Thus, bioavailability of free IGF-1 increases, which could mediate carcinogenesis in presence of other mediating factors. Should this hypothesis test positive, then it would be show insights to the role of insulin and insulin resistance in carcinogenesis.

A pilot study on 63 subjects was conducted to test this hypothesis (unpublished data). A trend towards significance of association between insulin resistance/sensitivity and IGF-1 levels was seen. The trend was stronger in patients below the age of 50. Insulin resistance and associated derangements are largely attributed to genetic factors [26,27]. Thus, advanced age could be a confounder for this line of investigation. The study shall be conducted on cancer patients, below the age of 50 years, with solid tumors (carcinomas). The study also includes a control group consisting of subjects from normal population. Various associations between IGF-1 and insulin resistance/sensitivity shall be determined by suitable statistical methods. However, the precise role of IGF-1 and the extent of influence of IGF-1 system on carcinogenesis cannot be determined in this study

This study could add to the knowledge and insights into insulin's relationship with IGF-1 system and insulin's role in carcinogenesis. The role of insulin and insulin resistance would be an interesting subject in cancer prevention and treatment.

## List of abbreviations

**IGF: **Insulin growth factor

**IGFBP: **Insulin growth factor binding protein

**FBS: **Fasting blood sugar

**GOD-POD: **Glucose oxidase – peroxidase

**RIA: **Radioimmunoassay

**IRMA: **Immunoradiometric assay

**HOMA-IR: **Homeostasis model assessment-insulin resistance

**SGA: **Subjective global assessment

**SPSS: **Satastical Package for Social Sciences

## Competing interests

The author(s) declare that they have no competing interests.

## Authors' contributions

**KH: **Co-investigator, Manuscript drafting, revision and finalizing for intellectual content before final approval

**MD: **principal investigator, concept of the study and intellectual content.

**HM: **Co-investigator, Manuscript drafting and study concept.

All authors have read and approved the final version of the manuscript.

## Pre-publication history

The pre-publication history for this paper can be accessed here:


